# Thresholding of auditory cortical representation by background noise

**DOI:** 10.3389/fncir.2014.00133

**Published:** 2014-11-10

**Authors:** Feixue Liang, Lin Bai, Huizhong W. Tao, Li I. Zhang, Zhongju Xiao

**Affiliations:** ^1^Department of Physiology, School of Basic Medicine, Southern Medical University, GuangzhouGuangdong, China; ^2^Zilkha Neurogenetic Institute, Keck School of Medicine, University of Southern CaliforniaLos Angeles, CA, USA

**Keywords:** auditory cortex, background noise, tonal receptive field, loose-patch recording, frequency tuning, intensity tuning, fast-spike inhibitory neuron

## Abstract

It is generally thought that background noise can mask auditory information. However, how the noise specifically transforms neuronal auditory processing in a level-dependent manner remains to be carefully determined. Here, with *in vivo* loose-patch cell-attached recordings in layer 4 of the rat primary auditory cortex (A1), we systematically examined how continuous wideband noise of different levels affected receptive field properties of individual neurons. We found that the background noise, when above a certain critical/effective level, resulted in an elevation of intensity threshold for tone-evoked responses. This increase of threshold was linearly dependent on the noise intensity above the critical level. As such, the tonal receptive field (TRF) of individual neurons was translated upward as an entirety toward high intensities along the intensity domain. This resulted in preserved preferred characteristic frequency (CF) and the overall shape of TRF, but reduced frequency responding range and an enhanced frequency selectivity for the same stimulus intensity. Such translational effects on intensity threshold were observed in both excitatory and fast-spiking inhibitory neurons, as well as in both monotonic and nonmonotonic (intensity-tuned) A1 neurons. Our results suggest that in a noise background, fundamental auditory representations are modulated through a background level-dependent linear shifting along intensity domain, which is equivalent to reducing stimulus intensity.

## Introduction

Natural acoustic signals are often accompanied with various types of background noise. Extracting sounds with significance from the competing environment imposes a complex challenge for listeners. Noise at high levels is usually detrimental to auditory perception, and can even lead to transient or permanent hearing loss (Berglund et al., 1996; Henderson and Salvi, 1998; Smith and Davis, 1999). Studies in the human have demonstrated that continuous background noise would both suppress the strength of sensory-evoked auditory responses at several different stages of the auditory pathway and reduce the discriminability in auditory behavior tests (Hari and Mäkelä, 1988; Martin et al., 1997; Whiting et al., 1998; Burkard and Sims, 2002; Morita et al., 2006; Billings et al., 2009). These effects are shown to be dependent on the noise level. On the other hand, there are studies suggesting that soft background noise would increase the amplitude of auditory evoked responses and improve the ability of signal detection (Galambos and Makeig, 1992; Zeng et al., 2000; Ries, 2007; Kishon-Rabin et al., 2008; Alain et al., 2009). Therefore, background noise might have bidirectional effects on auditory perception depending on the noise level.

**Figure 1:**
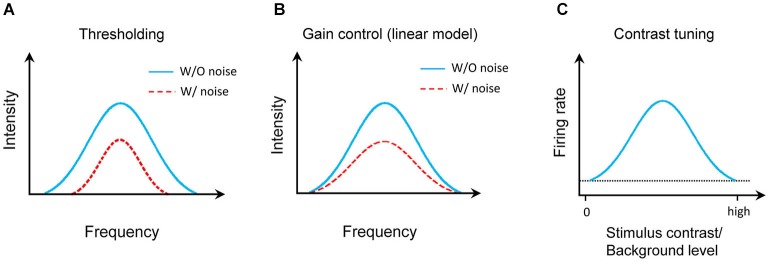
**Models of auditory cortical representation by background noise**. **(A)** Thresholding model. Blue solid line indicates the TRF for tone response in noise-free condition and red dotted line presents the TRF with background noise. **(B)** Linear gain control model. **(C)** Contrast tuning model. The horizontal dotted line shows the spontaneous firing rate of the model neuron.

The effect of noise background on the auditory cortical representation has been extensively studied in animal models. It is generally agreed that background noise does not change the auditory selectivity of cortical neurons, e.g., frequency tuning (Ehret and Schreiner, 2000), the temporal patterns (Zhou and Wang, 2010), sound-source location (Brugge et al., 1998). However, three different models were previously proposed to describe the effect of background noise (Figure 1). First, the background noise increases the responding sound intensity threshold (Figure 1A; Phillips, 1985, 1990; Phillips and Cynader, 1985; Phillips and Hall, 1986; Phillips and Kelly, 1992; Ehret and Schreiner, 2000). This was mostly based on the responses to testing tone stimuli at characteristic frequency (CF). Second, a linear gain control model was recently proposed from the study on the auditory cortex in anesthetized ferrets (Figure 1B; Rabinowitz et al., 2011, 2013). Thirdly, in awake marmoset, it was found that auditory cortical neurons were tuned to stimulus/background contrast (Figure 1C; Barbour and Wang, 2003). We revisited this issue by focusing on the frequency-intensity tonal receptive field (TRF), one of the most fundamental functional properties of auditory neurons. In addition, we also systematically characterized how different types of auditory cortical neurons (e.g., excitatory or inhibitory, monotonic or nonmonotonic neurons) respond in various background noise levels.

## Materials and methods

### Animal preparation

All experimental procedures used in this study were approved under the Animal Care and Use Committee of Southern Medical University. Experiments were carried out in a sound-attenuation booth, as previously described (Zhou et al., 2012a). A total of 55 adult female Sprague-Dawley rats (about 2 months old and weighing 200–250 g) were involved in the experiment. Animals were anesthetized with urethane (45 mg/kg; i.p.) and fixed with a custom-made holding apparatus that left the ears free. Body temperature was maintained at 37°C with a feedback temperature controller (69001, RWD). Right A1 was exposed via a craniotomy-duratomy (~2 mm diameter) and the ipsilateral (right) ear canal was plugged. The location of the craniotomy was determined stereotactically (5 mm posterior and 4 mm lateral from the bregma). During surgery and recording, the exposed cortex was moistened with a pre-warmed artificial cerebrospinal fluid (ACSF; in mM: NaCl, 132; NaHCO_3_, 20; KCl, 2.5; NaH_2_PO_4_, 1.2; MgSO_4_, 1.1; CaCl_2_, 2; HEPES, 3; glucose 15).

Free-field stimuli were generated digitally at 200 kHz using TDT System 3 (Tucker-Davis Technologies), and delivered through a calibrated electrostatic speaker (ES1, Tucker-Davis Technologies) located 10 cm lateral to the contralateral (left) ear. Multiunit recordings was carried out with parylene-coated tungsten microelectrodes (2 MΩ, FHC) at 500–600 µm below the pial surface to premap the auditory cortex and to locate A1. Pure tones (2–64 kHz at 0.1 octave intervals, 50-ms duration, 5-ms ramp) at eight 10 dB-spaced sound intensities (0–70 dB sound pressure level, SPL) were applied. Electrode signals were amplified by TDT System 3, band-pass filtered between 300 and 1000 Hz and then thresholded by BrainWare software to extract the spike time. The preliminary TRF was plotted online by BrainWare software to identify the CF of the recording site, and A1 location was determined by the anterior-posterior tonotopic gradient (from high to low frequency) (Zhou et al., 2012a; Li et al., 2013). The anterior auditory field (AAF) with reversed tonotopic gradient was also mapped to confirm the border of A1 and AAF.

### *In vivo* cell-attached loose-patch recording

After premapping of A1, cell-attached recordings (Zhou et al., 2010, 2012b; Zhang et al., 2011) were obtained from neurons located at 450–700 µm beneath the cortical surface, corresponding to layer 4 of the auditory cortex. Agar prepared in the ACSF (3.5%) was applied to reduce cortical pulsation during recording. Pipette (1–2 µm tip diameter, 7–9 MΩ of impedance, pulled with Sutter P2000) was filled with the ACSF solution. Spike current was recorded under voltage-clamp mode by an Axopatch 700B amplifier (Axon Instruments), with the baseline current adjusted to be near zero. Loose seal (50–500 MΩ) was made between cell and pipette, allowing spikes only from the patched cell to be recorded. Signals were filtered at 2 kHz with a sampling rate of 20 kHz. Spike shapes were sorted online by custom-developed MATLAB software (Mathworks), which enabled us to distinguish between regular-spiking and fast-spiking neurons. To search for auditory responsive A1 neurons, a 50-ms long white noise burst was presented at a rate of 2 Hz, while the electrode advanced dorsoventrally through the auditory cortex. After the formation of loose seal, TRF was mapped by applying 408 tones at different frequencies and intensities without background noise. The TRF of spike responses was plotted online with BrainWare software. The recording specifically focused on middle frequency representing regions of A1, which could be clearly distinguished with low-frequency nonmonotonic region (NM region) in ventral auditory field (VAF) reported in the previous study of rat A1 (Wu et al., 2006). Only neurons with distinct classical V-shaped TRFs were considered in this study. For monotonic neurons, CF responses at the highest testing intensity will be at least more than 90% of the maximum response (Wu et al., 2006), while the nonmonotonic neuron exhibits decreased spike responses (less than 70% of the maximal responses) at highest intensity levels after reaching a response peak. The percentage of recorded nonmonotonic cells was 6% in current study, which is consistent with previous studies in rats (Polley et al., 2004; Wu et al., 2006). The presumable inhibitory neurons were identified based on their fast-spike shape as previously described (Wu et al., 2006, 2008; Zhou et al., 2010, 2012a; Sun et al., 2013). The chance for obtaining a fast-spiking neuron is about 10%.

### Auditory stimulation

The continuous background noise covered the frequency range from 1 to 32 kHz (wide band) was applied at different levels from 0–48 dB SPL at 12 dB step. For each test, the continuous background noise was set at one fixed level, and tone stimuli (50 ms) at different frequencies or intensities were presented on top of the noise in a pseudo-random sequence at 2 Hz. Test on each noise level was repeated for 5–10 times. When the background noise was switched a different intensity level, a 10-s adaptation time was followed before a new set of tone stimuli were presented.

To examine intensity-dependent responses, tones at the CF of the cell with intensities varied in 0–90 dB SPL at 3 dB interval were applied. To test frequency-dependent responses, tone bursts at 51 different frequencies spaced between 2–64 kHz at 0.1 octave interval and a fixed intensity at 60 dB SPL were used. To examine the entire TRF, the recorded neuron was tested by tone bursts of various frequencies and intensities accompanied with continuous background noise at 0 or 36 dB SPL.

### Data analysis

With cell-attached recordings, spikes can be faithfully recorded because their amplitudes are large, normally higher than 100 pA compared to the <5 pA baseline fluctuation. Tone-driven spikes were identified within a 0–50 ms time window after the onset of the tone stimuli. The average evoked spike number for each stimulus was displayed in a pseudo-color map with custom-made software written in MATLAB. The TRF was smoothed by using cubic spline interpolation algorithm. The boundary (envelope) of TRF (i.e., frequency-intensity tuning curve) was determined based on the continuity of tone-evoked responses along the frequency and intensity domain, and was defined at the level of 30% of maximum spike response. The minimum threshold of TRF was defined as the tone intensity at the tip of the frequency-intensity tuning curve. The CF of the recorded neuron was set as the tone frequency or the logarithmic center of the frequency range at the minimum intensity threshold of TRF. The intensity threshold for CF tone in each noise condition was defined as the lowest tone intensity at which reliable spike responses were evoked in more than 30% of repetitions, and the evoked responses should be larger than two times SD of the baseline fluctuation.

## Results

Cell-attached recordings were obtained from 84 neurons in the A1 of total 55 rats, located at 400–700 µm below the pia, corresponding to layer 4 and upper layer 5 of the auditory cortex. We first mapped the spike frequency-intensity TRF of each recorded cell (see Section Materials and Methods). The CF and monotonicity of the cell were determined based on the reconstructed TRF. Excitatory or inhibitory cell type was categorized based on the shape of spike waveform (see Section Materials and Methods). Together, 73 monotonic excitatory neurons, 5 non-monotonic excitatory neurons and 6 inhibitory neurons were recorded. To examine changes of frequency and intensity representation under continuous noise background, neurons were tested with different tone-in-noise protocols (see Section Materials and Methods).

## Noise-level dependent linear shift of intensity threshold in excitatory monotonic neurons

**Figure 2:**
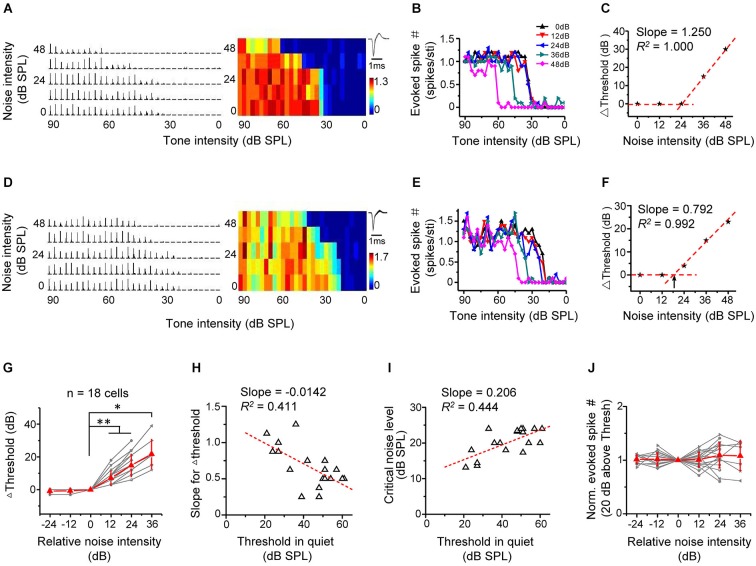
**Background noise linearly shifts intensity threshold of excitatory monotonic neurons. (A)** Left, PSTHs of responses of an example excitatory monotonic neuron to tones of the cell’s characteristic frequency (CF) at different tone intensities (0–90 dB at 3 dB step) and under different noise levels (0–48 dB at 12 dB step). Right, corresponding response color map. Color represents the evoked spike number by tones. Inset, average spike waveform. **(B)** Plot of evoked spike number vs. tone intensity for the same cell. Color represents different background noise levels. Note that the intensity threshold for tone evoked responses shifts toward higher values with increasing noise levels. **(C)** Change of intensity threshold (compared to noise-free condition) plotted against noise intensity for the same cell. The datapoint (0, 0) was derived by comparing the intensity threshold obtained in 0-dB (SPL) noise background with the minimum threshold of TRF in noise-free condition. The tilted red dotted line is the linear fit of data points above zero. Its slope represents the shift rate for Δthreshold. **(D–F)** Another example cell. Data are presented in the same manner. Arrow in **(F)** points to the critical noise level. **(G)** Change of intensity threshold plotted against the relative noise intensity. Gray represents individual neurons. Red represents mean ± SD (*n* = 18 cells). ^*^p < 0.05; ^**^p < 0.01, Wilcoxon signed-rank test. **(H)** The slope for Δthreshold plotted against the intensity threshold in 0 dB noise. Red dash line represents the best-fit linear regression line (*p* < 0.01). The slope and *R*^2^ are marked. **(I)** The critical noise level plotted against the intensity threshold in 0 dB noise. Red dash line represents the best-fit linear regression line (*p* < 0.01). **(J)** Normalized evoked spike number (at 20 dB above the intensity threshold) plotted against the relative noise level. No significant difference was detected (*p* > 0.05, Wilcoxon signed-rank test).

We first investigated the effect of background noise on intensity-dependent responses of A1 neurons. We applied tones of the CF of the recorded cell at different intensities, which were embedded in continuous background noise. The noise levels were set from 0 to 48 dB SPL at a 12 dB step. The spike response levels at different tone intensities were measured under conditions of increasing noise levels (Figures 2A–F for two example excitatory cells). The tones evoked significant spike responses above a certain threshold, which increased with further increasing tone intensities and quickly reached a saturating level (Figures 2B,E). The response-intensity functions of the two example cells (Figures 2B,E) indicated that they were monotonic neurons (Suga and Manabe, 1982; Wu et al., 2006). The intensity response regions progressively shifted towards higher values when the noise was above a certain level (Figures 2B,E). We plotted the relative intensity threshold (i.e., Δthreshold) in reference to the minimum threshold of TRF obtained in noise-free condition as a function of noise level (Figures 2C,F). The noise background with 0 dB SPL level showed no impact on the Δthreshold at all, so we refer to the condition with 0-dB noise as “in quiet”. The plotting showed more clearly that above a certain noise level, intensity threshold shifted toward higher values at a relatively constant rate with increasing noise levels, manifested by a good linear fitting of the data above this “critical” noise level (Figures 2C,F). We determined the critical noise level as the X-intercept of the linear regression line (shown by the arrows in Figure 2F). This method simulated a continuous variation of noise levels and therefore could extract a definite minimum noise level for an effective shifting of intensity threshold.

In all 18 similarly analyzed cells, there was a strong linear dependence of Δthreshold on noise levels above the critical noise level (*R*^2^ > 0.960, *p* < 0.01). The critical noise level for excitatory monotonic neuron was all >12 dB SPL (20.2 ± 3.8 dB SPL). To summarize the results, the testing noise intensity closest to the determined critical noise level was set as zero, and noise intensities were transformed into relative intensities in reference to this intensity level. The Δthreshold–relative noise intensity functions of different cells were then aligned and averaged (Figure 2G). The population data showed that intensity threshold remained constant (i.e., Δthreshold = 0) when the relative noise level was <0 and then increased more or less linearly when the relative noise level was >0 (Figure 2G). On individual-cell basis, the slope of linear fitting of threshold shifts negatively correlated with the intensity threshold in the quiet condition (Figure 2H; *R*^2^ = 0.411, *p* < 0.01), indicating that the lower the intrinsic threshold of the cell, the faster could the threshold be shifted toward higher values by increasing noise levels. In addition, the critical noise level positively correlated with the intrinsic threshold (Figure 2I, *R*^2^ = 0.444, *p* < 0.01). Together these data suggest that the detection thresholds are shifted proportionally with increasing background noise intensity levels. Finally, we examined the effect of noise background on the magnitude of intensity responses. Due to the up-shifting of intensity threshold under noise background, the range of effective tone intensities was reduced (Figures 2B,E). However, the average response amplitude within a small intensity range at 20 dB above the intensity threshold remained relatively stable across noise levels (Figure 2J). This suggests that background noise had little impact on intensity responses within equivalent effective intensity ranges above the noise-dependent threshold. Thus, for monotonic excitatory neurons, it appears that background noise simply modulates where the threshold is.

## Narrowing of frequency tuning without changes of frequency preference

**Figure 3:**
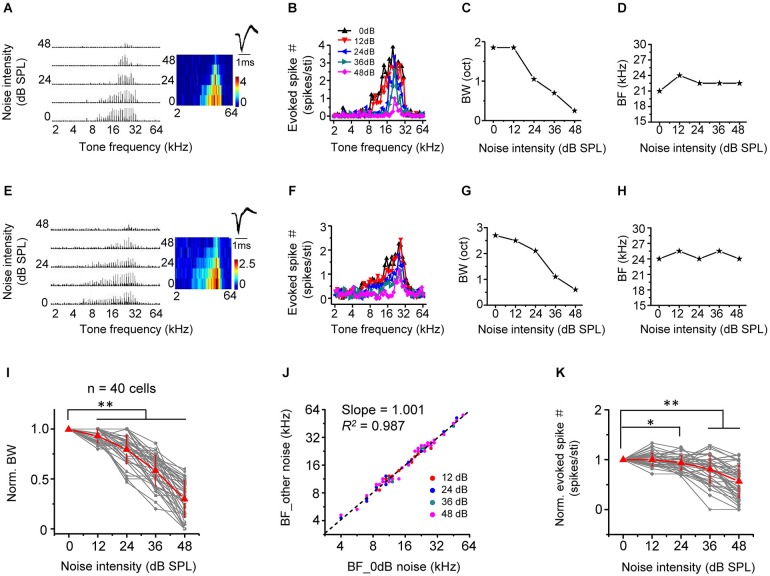
**Background noise narrows frequency tuning bandwidth. (A)** Left, PSTHs of responses of an example excitatory monotonic neuron to tones of 60 dB SPL at different frequencies (2–64 kHz) under different noise levels. Right, corresponding color map. Inset, spike waveform of the cell. **(B)** Evoked spike number at different tone frequencies for the same cell. Colors represent different noise levels. **(C)** Frequency tuning bandwidth (BW) plotted against noise intensity. **(D)** Best frequency (BF) at the tone intensity of 60 dB SPL plotted against noise intensity. **(E–H)** Another example neuron. Data are displayed in the same manner. **(I)** Normalized frequency tuning bandwidth (by the value under 0 dB noise) plotted against noise intensity (*n* = 40 cells). Red represents mean ± SD. ^**^ *p* < 0.01, Wilcoxon signed-rank test. **(J)** Best frequency under >0 dB noise vs. BF under 0 dB noise. Black dash line represents the best-bit linear regression line (*p* < 0.001). **(K)** Average spike number evoked by an effective tone (normalized by the value under 0 dB noise) plotted against noise intensity. ^*^p < 0.05; ^**^p < 0.01, Wilcoxon signed-rank test.

We next examined the effect of noise level on frequency processing properties of A1 neurons, by applying tones of various frequencies and at a fixed intensity of 60 dB SPL in the background of continuous noise. As shown by two example excitatory cells (Figures 3A–H), as noise level increased, the total range of effective tone frequencies (i.e., frequency tuning range) was progressively reduced (Figures 3B,F), suggesting that frequency tuning was narrowed. This is more clearly shown by the plot of frequency tuning bandwidth as a function of noise intensity (Figures 3C,G,I). At the highest testing noise level, tuning bandwidth was reduced by 69.8 ± 18.6% (*n* = 40 cells; Figure 3I, 0 dB vs. 48 dB: *p* < 0.01, Wilcoxon signed-rank test). Nevertheless, the best frequency (BF, defined as the tone frequency evoking the maximum response) was essentially unchanged under different noise conditions (Figures 3D,H,J, slope = 1.001, *R*^2^ = 0.987, *p* < 0.001). This indicates that background noise does not disrupt frequency preference of A1 neurons. Finally, the average response amplitude evoked by an effective tone stimulus was depressed in high-level background noise (Figure 3K, 0 dB vs. 48 dB: *p* < 0.01, Wilcoxon signed-rank test).

## Predictable changes of frequency tuning in noise conditions

**Figure 4:**
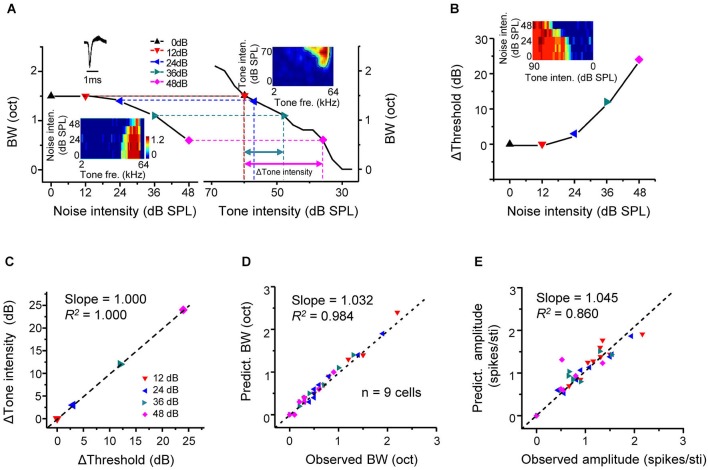
**Predictable changes of tone responses under background noise. (A)** Left, tuning bandwidth at tone intensity of 60 dB SPL plotted against noise intensity for an example excitatory monotonic neuron. Inset, color map for the frequency-dependent responses of the cell under different noise levels. Right, tuning bandwidths at different tone intensities (under 0 dB noise) for the same cell. Inset, color map for the frequency-intensity tonal receptive field (TRF) of the cell. Colored symbols mark tone intensity levels that would generate the same tuning bandwidth as the 60 dB tones under noise conditions. Colored arrows label the Δtone_intensity (relative to 60 dB SPL) needed to generate the same decrease in bandwidth as that produced by a certain increase in noise level. **(B)** Shift of intensity threshold plotted against noise intensity for the same cell. Inset, color map for its intensity-dependent responses under different noise levels. **(C)** The Δtone_intensity has a good correspondence with the shift of intensity threshold by the noise level (relative to 0 dB noise) that produces the same decrease in tuning bandwidth. Black line represents the best-fit linear regression line (*p* < 0.001). **(D)** Tuning bandwidths (60 dB tones, under different noise levels) predicted from the TRF (0 dB noise) by examining tone intensities shifted from 60 dB SPL by Δthreshold, plotted against the experimentally observed bandwidth (BW) under the noise level that produces the Δthreshold. *N* = 9 excitatory monotonic neurons (four data points per cell). Black dash line is the best-fit linear regression line (*p* < 0.001). **(E)** Average evoked spike number per tone stimulus under noise conditions predicted from the TRF (0 dB noise) plotted against the observed response amplitude (*p* < 0.01).

We have shown above that intensity-dependent responses were shaped by background noise in a linear manner. If the noise-induced changes of frequency-dependent responses are resulted in this linear shift of intensity responses, it is reasonable to assume that the impact of noise is equivalent to reducing sensory signals. To test this idea, frequency tuning bandwidths under different noise levels (Figure 4A, left) were plotted side-by-side with tuning bandwidths at different tone intensities extracted from the TRF of the cell in quiet (Figure 4A, right). Tone intensity in quiet producing the same tuning bandwidth as in a noise condition was identified (Figure 4A, dotted vertical lines). Through this comparison, a certain reduction of tuning bandwidth caused by an increase of noise level (in reference to 0 dB noise) could be transformed into a corresponding decrease of tone intensity in quiet (Δtone intensity, in reference to 60 dB SPL). In the same cell, the shift of intensity threshold in dependence of noise level was also tested (Figure 4B). The shift of intensity threshold in a noise condition matched well with the Δtone intensity at the same noise level (Figure 4C). In another word, the noise impact on A1 responses is equivalent to lowering the level of sensory stimulation in quiet.

From above, we speculate that response properties under noise background may be predictable from the TRF in quiet by correcting the level of tone stimulation according to the noise level. We tested the conformity of the experimentally observed and predicted response parameters, such as frequency tuning bandwidth and average response amplitude. The predicted tuning bandwidth and response amplitude for a given noise level were extracted from the TRF in quiet, after correcting tone intensity according to the noise-level dependent threshold change. For example, the frequency tuning bandwidth at 36-dB noise level (1.1 octave) was measured from the frequency tuning map composed of responses to 60-dB tones (Figure 4A, bottom-left inset). The intensity threshold of responses to CF-tone stimuli was increased by 12 dB under the same noise condition (Figure 4B). The predicted tuning bandwidth was then calculated from the TRF in quiet (Figure 4A, upper-right color map) at the tone intensity of 48 dB (60 dB minus 12 dB) SPL, which was also 1.1 octave. The same calculation of predicted tuning bandwidth was performed for each noise level. And the predicted and observed tuning bandwidths were compared in nine cells (Figure 4D). So were the predicted and observed average response amplitudes (Figure 4E). In both cases, we observed a strong conformity (Slope = 1.032, *R*^2^ = 0.984, *p* < 0.001 for Figure 4D, Slope = 1.045, *R*^2^ = 0.860, *p* < 0.05 for Figure 4E). These results demonstrate that the changes of frequency tuning properties and response strength in the presence of noise are highly predictable given the noise-level dependent threshold shift.

## Push-up effect on tonal receptive fields

**Figure 5:**
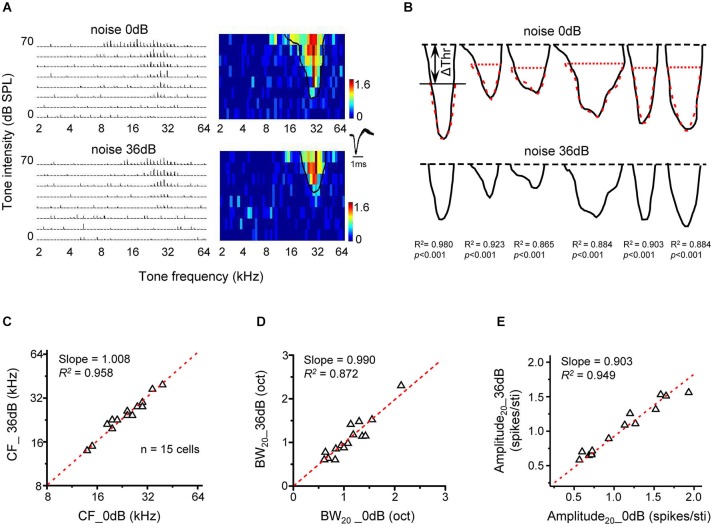
**Upward shifting of tonal receptive fields under noise conditions. (A)** PSTHs for tone evoked responses and the corresponding color map for an example cell under 0 dB (upper) and 36 dB (lower) noise backgrounds. The solid curve in the color map outlines the TRF boundary (determined at the level of 30% of maximum response level). **(B)** Tonal receptive field outlines for six neurons under 0 dB (upper) and 36 dB (lower) noise conditions. The top black dash line marks the highest tone intensity tested. The red dashed curve in the upper panel depicts the TRF outline under 36 dB noise (bottom) shifted downward by Δthreshold. The correlation coefficient for the top black and red curves is marked. **(C)** Characteristic frequency of the TRF under 36 dB noise is compared with that under 0 dB noise (*n* =15 cells). Red dash line is the best-fit linear regression line (*p* < 0.01). **(D)** Tuning bandwidth of TRF at 20 dB above the intensity threshold (i.e., BW_20_) under 36 dB vs. 0 dB noise. **(E)** Average response amplitude (evoked spike number per stimulus) at 20 dB above the intensity threshold under 36 dB vs. 0 dB noise.

The predictable changes of frequency tuning under noise conditions in present study suggest that noise exerts a simple up-shift effect on the entire TRF. To further test this idea, we compared TRFs mapped under two different noise conditions. In Figure 5A, TRFs recorded in quiet (upper panel) and in 36-dB noise (bottom panel) are shown for an example neuron. The solid black curve in the color map outlined the contour of the responsive frequency-intensity space (i.e., TRF), with its “tip” indicating the CF and minimum intensity threshold. In the presence of 36-dB noise, the minimum intensity threshold was elevated, so that the TRF in the testing frequency-intensity space appeared smaller (Figure 5A, bottom). Nevertheless, the shape of the TRF contour was not apparently changed except that the top part of the TRF in quiet was truncated. Figure 5B illustrates TRF contours in quiet (upper panel) and in the presence of 36-dB noise (lower panel) for six representative neurons. After shifting downward the TRF contour in 36-dB noise by the intensity level equivalent to Δthreshold in the same noise condition, we found that the TRF contours in quiet and in the presence of noise looked almost identical (Figure 5B, upper panel, compare the red dotted curve with the black curve). This suggests that noise simply “pushes up” the TRF without changing its shape. To demonstrate this point, we compared several TRF parameters in quiet and noise background. In a total of 15 cells analyzed in a similar manner, we found that the CF of TRF (Slope = 1.008, *R*^2^ = 0.958, *p* < 0.001, Figure 5C), as well as the frequency tuning bandwidth at the tone intensity of 20 dB above the “tip” of TRF (Slope = 0.990, *R*^2^ = 0.872, *p* < 0.001, Figure 5D) was essentially the same in the quiet and noise conditions. In addition, the average response amplitude at the tone intensity of 20 dB above intensity threshold was also similar between the quiet and noise condition (Slope = 0.903, *R*^2^ = 0.949, *p* < 0.001, Figure 5E). Therefore, noise does not change fundamental TRF parameters except that it shifts the entire TRF upward by Δthreshold.

## Linear shift of intensity tuning in nonmonotonic neurons

**Figure 6:**
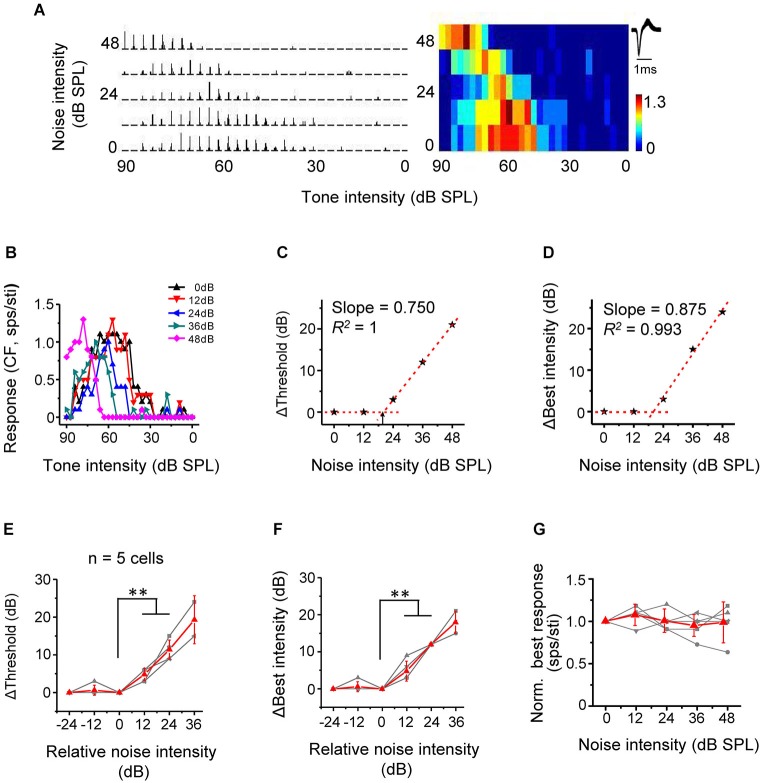
**Noise results in a linear shift of intensity tuning of nonmonotonic excitatory neurons. (A)** PSTHs for intensity-dependent responses of an example neuron to CF tones (left) and the corresponding color map (right). Note that the response level is low at both low and very high intensities. **(B)** Plot of response level against tone intensity for the same cell. **(C)** Change of intensity threshold (compared to 0 dB noise condition) plotted against noise level for the same cell. Arrow points to the critical noise level. **(D)** Change of best intensity (compared to 0 dB noise condition) plotted against noise level for the same cell. **(E)** Shift of intensity threshold as a function of noise intensity for five excitatory nonmonotonic neurons. Red displays mean ± SD. ^**^p < 0.01, Wilcoxon signed-rank test. **(F)** Shift of best intensity as a function of noise level. ^**^p < 0.01, Wilcoxon signed-rank test. **(G)** Normalized response amplitude at the best intensity under different noise levels.

Nonmonotonic neurons are powerfully influenced by inhibitory inputs and usually exhibit enclosed, narrow TRFs distinct from the V-shaped response areas of monotonic neurons (Suga and Manabe, 1982; Ojima and Murakami, 2002; Wang et al., 2002; Sutter and Loftus, 2003; Sivaramakrishnan et al., 2004; Wu et al., 2006, 2011). Although nonmonotonic (or intensity selective) neurons are abundant in the cat A1 (Phillips et al., 1995; Heil and Irvine, 1998), such neurons are relatively rare in the rodent A1 (Polley et al., 2004; Wu et al., 2006). In present study, we identified five nonmonotonic excitatory neurons based on their responses to CF tones at different intensities in the quiet condition (see Section Materials and Methods). The five nonmonotonic neurons were recorded from the middle-frequency representing region of A1, with their CFs lying between 20 kHz to 48 kHz, which were much different from the low-frequency NM region (Wu et al., 2006). We then tested their intensity-dependent CF-tone responses under different noise conditions. An example cell was shown in Figure 6A. In the quiet condition, the spike response increased initially with increasing tonal intensities above the threshold, reached a peak (i.e., at the best intensity), and then reduced to zero, forming a strongly nonmonotonic intensity tuning curve (Figure 6B, black). With increasing noise levels, the intensity threshold progressively shifted toward higher values (Figures 6B,C). Concurrently, the best intensity (intensity at which the maximum response was evoked) also progressively shifted toward higher values (Figures 6B,D). Summary of all nonmonotonic neurons further confirmed the observation. Similar to monotonic cells, noise when above a certain critical level shifted intensity threshold in a highly linear manner (Figure 6E). No significant difference in the rate of threshold shift was detected between nonmonotonic and monotonic neurons (*p* > 0.05, *n* = 5 and 18, Mann Whitney test). The best intensity was shifted in parallel, also in a highly linear manner (Figure 6F). The best intensity was elevated by a level similar to the increase of intensity threshold (*p* > 0.05, *n* = 5, Wilcoxon signed-rank test). Finally, the response amplitude at best intensity was unchanged across noise conditions (Figure 6G). This result, together with the parallel changes of intensity threshold and best intensity, supports the notion that noise simply shifts the entire intensity-tuning curve without changing the level of best intensity relative to intensity threshold. Therefore, noise also exerts a push-up effect on nonmonotonic responses. It is conceivable that when noise is strong enough, the nonmonotonic tuning curve can be truncated into a monotonic tuning curve by the upper bound of the testing intensity range (e.g., Figure 6B, purple).

## Up-shift of intensity and frequency tuning of inhibitory neurons

**Figure 7:**
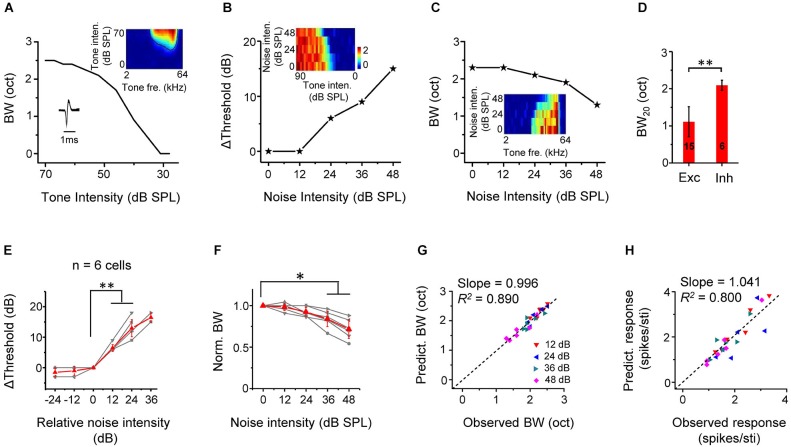
**Thresholding shift of inhibitory neuron responses by background noise. (A)** Tuning bandwidths at different tone intensities for an example inhibitory neuron. Inset, the color map for the TRF of the cell (upper) and its spike shape (lower). Note that the cell was a fast-spiking cell. **(B)** Shift of intensity threshold as a function of noise level for the same neuron. Inset, color map for its intensity-dependent responses to CF tones under different noise levels. **(C)** Tuning bandwidth as a function of noise level for the same cell. Inset, color map for its frequency-dependent responses to tones of 60 dB SPL under different noise levels. **(D)** Comparison of BW_20_ of TRF between inhibitory (*n* = 6) and excitatory (*n* = 15, same as in Figure 5D) cell groups. ^**^p < 0.01, Mann Whitney test. **(E)** Summary of shift of intensity threshold as a function of relative noise level (*n* = 6 inhibitory cells). ^**^p < 0.01, Wilcoxon signed-rank test. **(F)** Summary of normalized BW as a function of noise level. ^**^p < 0.05, Wilcoxon signed-rank test. **(G)** Bandwidth predicted from the TRF under different noise levels vs. BW experimentally observed for all the inhibitory cells. Black dash line is the best-fit linear regression line (*p* < 0.001). **(H)** Predicted response amplitude vs. that observed. Black dash line is the best-fit linear regression line (*p* < 0.01).

We identified six putative inhibitory neurons based on their narrow spike waveforms (Figure 7A, lower left inset). On average, the trough-to-peak interval of spike shape was 0.29 ± 0.03 ms for fast-spike inhibitory neurons, and 0.71 ± 0.15 ms for regular spiking neurons (*p* < 0.01, *n* = 6 and 40, Mann Whitney test). They were all monotonic cells, manifested by increasing tuning bandwidths with increasing tone intensities (Figure 7A). These six inhibitory cells exhibited relatively broad, U-shaped TRFs while excitatory cells had sharp, V-shaped TRFs, as verified by the significant broader tuning bandwidth of fast-spike neurons than the excitatory neurons (Figure 7D), consistent with the previous reports (Wu et al., 2008, 2011). Similar to excitatory cells, the threshold for CF-tone responses was linearly elevated by noise above a certain critical level (Figures 7B,E). The tuning bandwidth at the fixed tone intensity of 60 dB SPL was reduced by noise (Figures 7C,F). Using the same method to predict changes in response properties in noise for excitatory monotonic neurons (Figures 4D,E), we found in inhibitory neurons that there was a good correspondence between the predicted and observed tuning bandwidths (Figure 7G), as well as between the predicted and observed average response amplitude (Figure 7H). These results demonstrate that background noise also exert an up-shifting effect on TRFs following the linear shift of intensity thresholds of inhibitory neurons.

### Discussion

Low-level sound signals are undetectable in a noisy background, especially when the noise intensity is close to the threshold of subject recognition ability (Wang and Bilger, 1973; Phatak et al., 2008; Shetake et al., 2011). This is a well-established masking phenomenon in psychoacoustics. Some earlier studies have characterized the electrophysiological responses of different auditory cells along the auditory pathway to acoustic signals in the presence of continuous wide-spectrum noise. Those studies have relied on spike sorting to isolate single units, and have not examined the effect of noise on complete TRFs of individual cells (Costalupes et al., 1984; Ehret and Moffat, 1984; Phillips, 1985, 1990; Phillips and Cynader, 1985; Phillips and Hall, 1986; Ramachandran et al., 2000). In the current study, we applied cell-attached recordings to better isolate responses from single neurons and distinguish cell types based on spike shapes. By applying tones of various frequencies and intensities, we mapped the entire TRF of different types of neurons in A1 under varying noise conditions to provide a more complete description of the impact of noise background.

### Push-up effect of background noise on auditory cortical representation

Regardless of cell types, a general pattern of noise-induced elevation in the detection threshold is manifested. Along the intensity domain, noise can shift the intensity-dependent responses to a tone as an entirety, when its intensity is above a critical level. The shift of threshold is linearly dependent on the noise intensity above the critical level. Our results are consistent with a thresholding model, as suggested from previous studies (Phillips and Hall, 1986; Ehret and Schreiner, 2000).

Since the frequency tuning range to the tone stimuli of a fixed intensity is dependent on the background noise level (i.e., noise-variant), our results do not support a linear gain control model (Rabinowitz et al., 2011, 2013). This could be due to the difference in designing the sound stimulation. We have followed many previous studies with tone stimuli on top of a constant continuous background noise, while in the studies of Rabinowitz et al. (2011), or Barbour and Wang (2003), the background noise was under dynamic modulation. Our study did not exclude the potential tuning of auditory cortical neurons to various noise levels. Although recorded neurons in general exhibited reduced response level at higher background noise (or in other words, lower contrast), some individual neurons did exhibit different response-level profiles, e.g., with elevated responses even at high noise levels (Figure 3K). However, due to the large fluctuation of responses and limited repetition, this possibility remains to be further investigated in the future.

In addition, we should note that with the preserved shape of TRFs but increased intensity threshold, although frequency tuning as measured by the bandwidth at 20 dB above the threshold stayed the same, for the tone stimuli of the same intensity, the responding frequency range was reduced. In other words, the frequency selectivity should be increased under noise background. This in fact provided an explanation for the previous psychophysical observations that noise background can improve the ability of signal detection (Zeng et al., 2000; Ries, 2007; Kishon-Rabin et al., 2008).

### The rate of threshold shift

**Figure 8:**
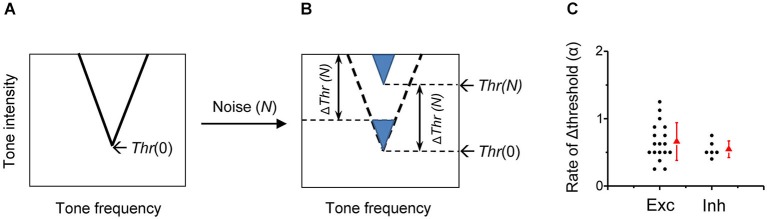
**Impacts of background noise on auditory cortical representation**. **(A)** Schematic drawing of a V-shaped TRF of an auditory cortical neuron in the absence of noise. The intensity threshold in quiet (*Thr*(0)) is marked. **(B)** In the presence of noise at the level *N*, the entire TRF is shifted upward by a level of *Thr*(*N*)-*Thr*(0) (i.e., *ΔThr (N)*), which is equivalent to lowering an equal level of tone intensity in quiet. **(C)** Comparison of the shift rate of intensity threshold (i.e., α value) between excitatory monotonic neurons (*n* = 18, same as in Figure 2) and inhibitory neurons (*n* = 6).

A simple formula is proposed to depict the transfer function for the noise-induced changes of detective intensity thresholds in A1 neurons. Figure 8A shows the schematic TRF of a typical A1 neuron in the frequency and intensity space in quiet condition, with its tip indicating the CF and minimum intensity threshold (*Thr*). When brought into a noise (at the level *N*) condition, the *ΔThr(N)* can be described by a threshold-linear function:

$$\Delta Thr(N)=\{\begin{array}{l} 0\;N\leq N_{0}\\ \alpha (N-N_{0})\;N>N_{0}\; \end{array}$$

As showed in the formula, below the critical noise level *N*_0_, noise has no effect on the intensity threshold. When the noise level is above *N*_0_, the shift of threshold at the noise level *N* is linearly related to the level of noise above *N*_0_. The value of *N*_0_ is 19.8 ± 4.2 dB SPL when averaged for all rat A1 neurons. The TRF of the neuron is shifted upward in the frequency and intensity space by *ΔThr(N)*, without changes in frequency preference or tuning sharpness (Figure 8B). The noise impact on A1 frequency-intensity responses is equivalent to lowering an equal level (i.e., *ΔThr(N)*) of sensory stimulation in quiet.

The strength of noise effects is reflected by the rate of noise-level-dependent threshold shift, which is defined as the increase of detection threshold per 1-dB increment of noise level. The rate of threshold shift has been computed for different sites along the auditory pathway. It has been shown that the noise-induced threshold shift is on average 0.61 dB for each 1-dB increment of background noise level in the auditory nerve of anesthetic cat (Costalupes et al., 1984), 0.80 dB/dB in the type I unit of the ventral cochlear nucleus and 1.05 dB/dB in type I ICC neurons of decerebrate cat (Ramachandran et al., 2000). Previous single-unit studies in barbiturate-anesthetized cat A1 show that the majority of neurons exhibit a threshold shift rate of close to 1 dB/dB, meaning that the increment of signal detection threshold closely matches the increment of noise level (Phillips and Cynader, 1985; Phillips, 1990). In the present study of urethane-anesthetized rat A1, the shift rate, also expressed by the slope of Δthreshold induced by noise increments (coefficient *α*), was on average 0.67 dB/dB in excitatory monotonic neurons, the predominant cell type in the rat A1 (Figure 8C). The variability of the rate however is relatively high among the cells, with rate values ranging from 0.28 to 1.25 dB/dB (Figure 8C). It’s possible that on top of the effects of noise on the mechanical threshold, the complex local circuits at each level of auditory processing modulate the threshold shift, leading to the diversity of shift rates among cortical cells. However, the species difference, the different anesthesia reagents or the recording methods may also contribute to the observed difference in shift rates in A1.

### Conformity among cell types

In this study, the general push-up effect of noise is universally observed for the three types of neuron in the rat A1: the monotonic excitatory neuron, nonmonotonic excitatory neurons, and fast-spiking inhibitory neuron. Neurons with nonmonotonic response-level functions, although only infrequently encountered, also exhibit a simple shift of response-level function. As a result, the best intensity of response-level functions shifts concurrently with the threshold. The rate of threshold shift is not different between monotonic and nonmonotonic excitatory neurons, consistent with previous reports in the ICC of anesthetized guinea pigs or decerebrate cats and A1 of anesthetized cat (Phillips, 1985; Rees and Palmer, 1988; Ramachandran et al., 2000). Whether neurons in other auditory NM regions, e.g., the NM region of rat (Wu et al., 2006), exhibit the same properties as described here remains to be investigated. Since the monotonicity or nonmonotonicity of the intensity response is determined by different excitatory and inhibitory processes, the comparable shift rates of thresholds between the two neuron types suggest that the mechanisms underlying the noise masking may exhibit an equivalent role on the excitatory and inhibitory events of tone-evoked response.

We specifically examined inhibitory neurons to test whether their responses are differentially modulated by background noise. The fast-spiking inhibitory neurons display more broadly tuned receptive fields compared with the sharp V-shaped receptive fields of excitatory neurons (Figure 7D), consistent with previous studies (Atencio and Schreiner, 2008; Wu et al., 2008; Li et al., 2014, but see Moore and Wehr, 2013). While in excitatory neurons the tuning width at 60-dB tone intensity was reduced in 48-dB noise by 69.8 ± 18.6% (Figure 3I), in inhibitory neurons it was only reduced by 28.2 ± 12.4% (Figure 7F). However, the threshold shift rate of inhibitory neurons is not significantly different from that of excitatory neurons (Figure 8C), although the variation of the rate appears smaller in the inhibitory cell group (0.015 for inhibitory cell; 0.079 for excitatory cell). That smaller reduction of frequency bandwidth in inhibitory neuron could be due to the broader shape of their TRFs. Therefore, background noise appears to exert a rather homogeneous influence on signal processing of all cortical neurons.

In summary, our data of single cortical neurons obtained by *in vivo* cell-attached recordings strongly demonstrate a thresholding effect of background noise, with the overall shape of the auditory receptive field largely preserved. Thus, the auditory circuits are designed and structured to be immune for noise intervention, which may ensure a more consistent auditory representation and processing in both quiet and noisy environments.

## Conflict of interest statement

The authors declare that the research was conducted in the absence of any commercial or financial relationships that could be construed as a potential conflict of interest.
